# Retraction Note: Enhanced antitumor efficacy in colon cancer using EGF functionalized PLGA nanoparticles loaded with 5-Fluorouracil and perfluorocarbon

**DOI:** 10.1186/s12885-026-16805-6

**Published:** 2026-08-22

**Authors:** Pingping Wu, Qing Zhou, Huayun Zhu, Yan Zhuang, Jun Bao

**Affiliations:** Department of Medical Oncology, Jiangsu Cancer Hospital & Jiangsu Institute of Cancer Research & The Affiliated Cancer Hospital of Nanjing Medical University, Nanjing, 210009 China

**Retraction Note: BMC Cancer 20, 354 (2020)**

**https://doi.org/10.1186/s12885-020-06803-7**

The Editors have retracted this article. After publication, concerns were raised regarding the images. Specifically, similarities were detected between the fourth and sixth panels of the second row (Liver) of Fig. 5A. The authors have not provided a response to these concerns. Therefore, the editors have lost confidence in the data and conclusions of this article.

Authors Pingping Wu, Qing Zhou, Huayun Zhu, Yan Zhuang, and Jun Bao did not respond to correspondence from the Publisher about this retraction.

